# Stomata as the Main Pathway for the Penetration of Atmospheric Particulate Matter Pb into Wheat Leaves

**DOI:** 10.3390/toxics13030185

**Published:** 2025-03-01

**Authors:** Ke Zhang, Yujing Liang, Chuang Ma, Haopeng Guo, Fuyong Liu, Aihua Gao, Nan Liu, Hongzhong Zhang

**Affiliations:** 1 School of Material and Chemical Engineering, Zhengzhou University of Light Industry, Zhengzhou 450000, China; 2017093@zzuli.edu.cn (K.Z.); yujingliang3322@163.com (Y.L.); haopengguo@126.com (H.G.); liu.fyong@gmail.com (F.L.); pirlo2008@zju.edu.cn (N.L.); zhz@zzuli.edu.cn (H.Z.); 2 Collaborative Innovation Center of Environmental Pollution Control and Ecological Restoration, Zhengzhou 450000, China; 3 Zhongyuan Ecological Environment Technology Innovation Center (Henan) Co., Ltd., Zhengzhou 450000, China

**Keywords:** wheat leaves, atmospheric particulate matter Pb, stomata, cuticle, absorption pathway

## Abstract

The absorption of atmospheric particulate matter lead (APM-Pb) by wheat leaves is the primary source of Pb in wheat grains, yet the mechanisms of how wheat leaves absorb Pb remain unclear. In this study, spraying Pb(NO_3_)_2_ (Treatment T1) and spraying PbS (Treatment T2) were used as soluble and insoluble Pb, respectively, to evaluate the primary pathways of APM-Pb absorption by wheat leaves, as well as the translocation and accumulation patterns of Pb within the wheat plant. The results showed that both soluble and insoluble Pb can be absorbed by wheat leaves. Compared to the control group (CK), the treatment of T1 and T2 significantly increased Pb concentration in both leaves and grains, as well as the Pb accumulation rate in grains (*p* < 0.05). Scanning electron microscopy–energy dispersive spectrometry (SEM-EDS) technology visually confirmed the distribution of particulate Pb in the stomatal region, demonstrating that solid-state Pb can penetrate the leaves through stomata. From the greening stage (GS) to the late filling stage (FS2), the leaves’ cell sap contained the highest proportion of Pb, indicating that Pb within the cell sap possesses the greatest capacity for translocation. Concurrently, a significant increase in grain Pb concentration during this period indicated that the migration of Pb to cell sap after penetrating the leaves is subsequently translocated to the grains (*p* < 0.05). Compared to the jointing stage (JS), the proportion of the ethanol and water extraction states of Pb significantly decreased in FS2 (*p* < 0.05), indicating that Pb is more readily translocated to the grains during this period. Moreover, in FS2, Pb concentration in leaves and grains in the T2 treatment reached 76.5% and 63.9% that of T1, respectively. Since PbS can only be absorbed through stomata, it can be inferred that stomata are the primary pathway for wheat leaves to absorb APM-Pb. Therefore, Pb absorbed through the stomatal pathway and accumulated in the cell sap fraction is most likely to be translocated to the grains during the filling stage. This study provides new insights into the mechanisms of Pb absorption and translocation in wheat, emphasizing the critical role of stomata in the uptake of APM-Pb. It offers a new direction for breeding wheat varieties resistant to APM-Pb pollution, which is of significant importance in agricultural practices aimed at reducing heavy metal contamination in crops.

## 1. Introduction

Wheat is not only a primary source of carbohydrates for human consumption but also provides a variety of nutrients, including protein, vitamins, and minerals. It has several advantages, such as strong adaptability to processing, ease of storage and transportation, and significant economic benefits, making it an indispensable part of the human diet. Additionally, wheat is a crop that boasts strong adaptability to soil and climate conditions. It also has low planting costs and is easy to manage, which is why it is widely cultivated [1]. Wheat-growing areas are mainly concentrated in China, India, and Russia in Asia, the European Union in Europe, the United States and Canada in the Americas, and Australia in Oceania. These countries and regions account for the majority of global wheat production, ensuring global food security and market demand [2]. China is the world’s largest producer and consumer of wheat. Its cultivation areas spread throughout the country, mainly in the northern regions, including areas south of the Great Wall, east of the Liupan Mountains, and north of the Qinling Mountains and the Huai River.

However, soil contamination has become a global issue, posing a potential threat to the growth and safety of wheat [3]. Reports from the Ministry of Land and Resources of China and the Ministry of Environmental Protection of China indicate that approximately 4 million hectares of arable land (accounting for 2.9% of arable land in China) are moderately or highly polluted by contaminants (Bulletin on National Survey of Soil Contamination). Currently, more than 20,000,000 acres of agricultural land in China—accounting for 25% of the total farmland area—are polluted by toxic elements such as lead (Pb), cadmium (Cd), chromium (Cr), tin (Sn), and zinc (Zn) [4]. Soil Pb contamination is a widespread issue within China’s agricultural settings [5,6,7,8], and the average concentration of Pb in surface soil is 35.9 ± 0.21 mg·kg^−1^ [9]. The causes of soil Pb pollution are related to electronic waste recycling, industrial emissions of wastewater, and exhaust gasses. In this regard, exhaust gasses not only accumulate in the soil, causing Pb pollution, but can also directly contact plant leaves, leading to plant contamination [6,10]. China’s ambient air quality standards set an annual average concentration limit of 0.5 µg·m^−3^ for Pb and a quarterly average concentration limit of 1 µg·m^−3^. However, many areas fail to meet these standards due to the discharge of exhaust gasses, and hence, the impact of Pb from atmospheric deposition should not be underestimated [11].

As a vital food crop, wheat has the ability to absorb Pb from the soil through its roots, which potentially contaminates the grain and poses substantial health hazards to humans [8,12,13,14]. Studies on Pb contamination in wheat are mainly focused on the mechanisms of Pb translocation within the soil–wheat system [15,16,17,18]. However, Pb isotope-tracing techniques have been utilized to identify additional routes through which wheat can become contaminated with Pb [19,20], highlighting the influence of atmospheric particulate matter Pb (APM-Pb) on wheat. Historically, most studies on wheat Pb contamination have been conducted at sites with known Pb pollution [19,21]. However, it is significant to note that even in areas such as the North China Plain, which are distant from recognized Pb pollution sources, wheat grains still exhibit a notable tendency to accumulate Pb [19,22,23]. A survey by Wang et al. [20], which examined 50 farmlands in Henan and Hebei provinces, revealed a stark contrast: only 13% of the soil samples had excessive Pb levels, while a concerning 100% of the wheat grain samples did. This further indicates that soil is not the sole source of Pb contamination in wheat.

Pb typically penetrates wheat leaves through two primary avenues: root absorption of soil Pb and leaf absorption of APM-Pb from leaf surfaces [15,20,24,25]. This dual mechanism has directed research attention towards the influence of APM-Pb on wheat leaves. Studies have demonstrated that APM-Pb in direct contact with wheat leaves is the predominant source of Pb in wheat grains, with leaves during the early filling stage (FS1) being the primary contributors to grain Pb concentration [21]. Furthermore, Schreck et al. [7] showed that the concentration of heavy metals in crops, such as lettuce, which are cultivated in proximity to Pb smelters, increases with extended exposure times. Analogously, Zhang et al. [23] found that the absorption of APM-Pb through the leaves of winter wheat is a primary pathway for Pb contamination in grains [19,22]. Thus, to clarify the leaf absorption pathway of Pb, it is essential to investigate the mechanisms of Pb uptake by leaves, which will help reveal how Pb translocate to grains.

Leaf absorption of APM-Pb can translocate to other organs via stomatal and cuticular pathways, with the predominant mechanism being influenced by factors including the particle size and solubility of APM-Pb, the chemical composition of the leaf surface, and weather conditions [26,27]. Atmospheric Pb is predominantly present in fine particles, such as PM_2.5_, with the primary phases of PM-Pb (Pb, PbS, PbO, PbSO_4_, and PbO·PbSO_4_) being predominantly insoluble compounds [18]. In this study, Pb(NO_3_)_2_ solution and PbS suspension were used to simulate the soluble and insoluble components of APM, respectively. The quantity of Pb(NO_3_)_2_ that penetrated the leaves was considered to represent Pb that infiltrated through both the cuticle and stomata, whereas the quantity of PbS that penetrated the leaves was used to signify Pb that entered solely through the stomata. By comparing these two quantities, we were able to evaluate the contributions of the cuticle and stomata to the entry of APM-Pb into leaves.

Given the evidence that APM-Pb significantly contributes to Pb levels in wheat grains, and that leaves during the FS1 are major contributors to grain Pb [21,23], we hypothesized that the stomatal pathway is the primary route for wheat leaves to absorb APM-Pb. This hypothesis is based on the observation that APM-Pb, especially in the form of fine particles such as PM_2.5_ that are predominantly insoluble, are more likely to penetrate leaves through stomata due to their direct exposure to the atmosphere. To test this hypothesis, wheat leaves in the greening stage (GS) were subjected to Pb stress using Pb(NO_3_)_2_ solution and PbS suspension, which simulated the soluble and insoluble fractions of APM, respectively. This experimental approach enabled us to explore the main absorption pathways for APM-Pb by leaves, evaluate whether absorbed Pb can cross the leaf cell membranes, and ascertain whether it can be translocated to other plant organs. The hypothesis was further substantiated by analyzing the subcellular distribution of Pb in leaves, conducting chemical extractions of Pb from wheat leaves, and performing SEM-EDS analysis to visualize Pb distribution and translocation. These methods offer a comprehensive understanding of the role of the stomatal pathway in APM-Pb absorption and its impact on wheat grain contamination.

## 2. Materials and Methods

### 2.1. Study Area

**Figure 1:**
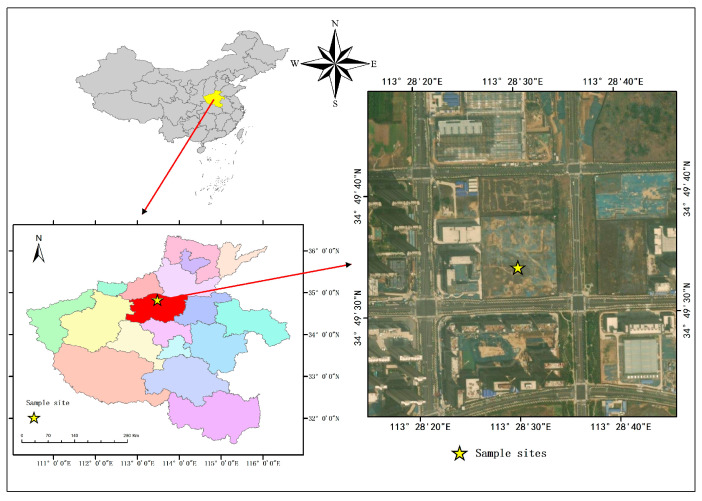
The location of the experiment.

The test field was established in Zhengzhou City, Henan Province, China (34°49′31.3647″ N, 113°28′53.4857″ E) (Figure 1), with a temperate continental monsoon climate and an average annual precipitation of 629.7 mm [28]. The soil type is cinnamon soil, and the total pH value, carbon, organic carbon, total nitrogen, ammonium nitrogen, available phosphorus, and total phosphorus concentrations are 8.36, 11.67 g·kg^−1^, 24.648 g·kg^−1^, 0.93 g·kg^−1^, 5.55 mg·kg^−1^, 22.22 mg·kg^−1^, 32.4 mg·kg^−1^, and 0.62 g·kg^−1^, respectively. The Pb concentration in soil is 6.10 mg·kg^−1^, which is lower than the environmental quality standard, and the Pb concentration of atmospheric precipitation in the study area is 78.50 mg·kg^−1^, indicating no significant Pb pollution in the study area.

### 2.2. Experimental Reagents

The particle size of PbS particles used in the experiments was below 300 mesh (Shanghai McLean Biochemical Technology Co., Ltd., Shanghai, China), with 78.4% of the particles having a particle size of less than 20 μm, 1.3% of the particles being larger than 40 μm, and 20.3% of the particles being in the range of 20–40 μm. The Pb(NO_3_)_2_ reagent used was of analytical purity.

### 2.3. Experimental Design

**Figure 2:**
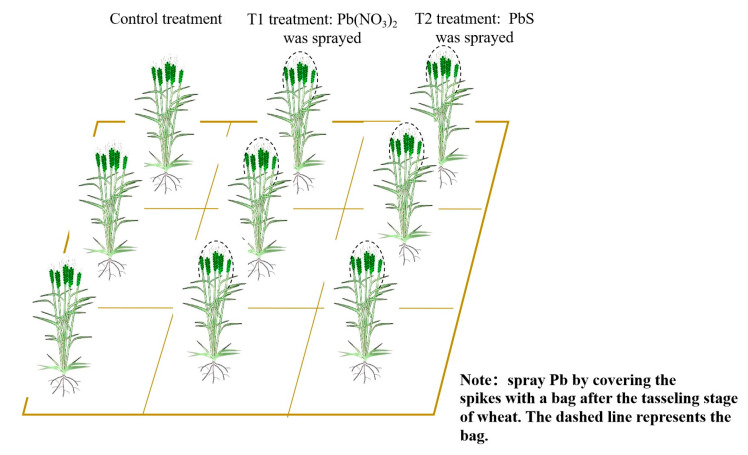
Diagram of the experimental design.

Wheat was sown on 22 October 2023 (Zhengmai 618), and normal field management was carried out during planting, with reference to local planting habits. During the GS (11 March 2024), nine sample squares with an area of 1 m^2^ were randomly selected in the wheat field and divided into three groups: one sprayed with Pb(NO_3_)_2_, one sprayed with PbS, and a control treatment. Spraying of 150 mL of 50 mg·L^−1^ PbS suspension and Pb(NO_3_)_2_ solution per square meter above the wheat in the corresponding sample plots was carried out from the GS, and Pb was sprayed by covering the spikes with a bag after the tasseling stage of wheat (Figure 2). The spraying treatments were carried out at intervals of 5 days for a total of 15 times, and the operation was adjusted appropriately in rainy and windy weather.

**Figure 3:**
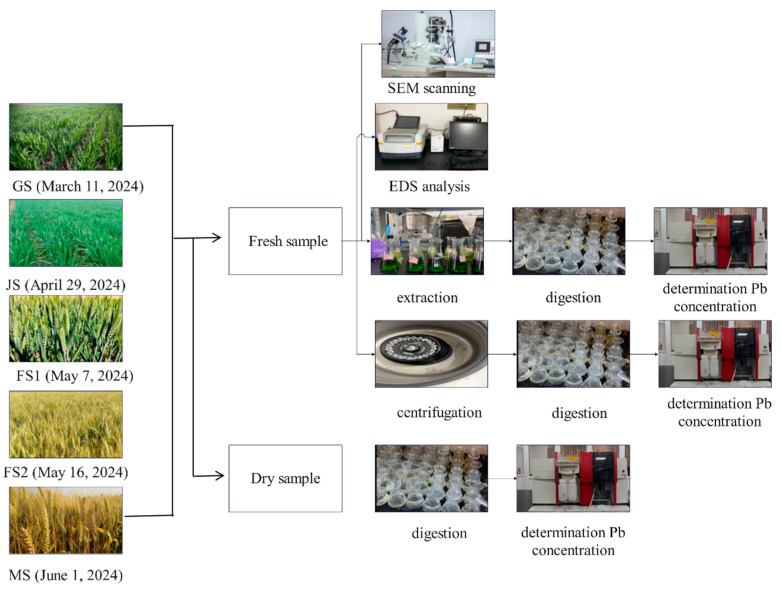
The diagram of the experimental process.

Samples were collected at GS (11 March), jointing stage (JS) (29 April), FS1 (7 May), late filling stage (FS2) (16 May), and mature stage (MS) (1 June). Thirty wheat plants were collected from each treatment in each period and divided into two parts: one was dried for the analysis of Pb concentration in various organs of wheat and the other fresh samples were used for the analysis of subcellular distribution of Pb, chemical extraction state analysis of Pb, and SEM-EDS analysis. See Figure 3 for details.

### 2.4. Sample Analysis

#### 2.4.1. Determination of Pb Concentration

Wheat samples were digested by the HNO_3_-HClO_4_-H_2_O_2_ method with an electric hot plate [29]. Specifically, 0.50 g of wheat sample was weighed into a triangular flask, and 8 mL HNO_3_, 2 mL HClO_4_, and 2 mL H_2_O_2_ were added sequentially. The sample was heated and digested on the following day, with the initial temperature set at 140–160 °C. After that, the temperature continued to rise and the sample was evaporated to obtain 1–2 mL of digestion solution, and then the electric hot plate was turned off. After cooling, the samples were diluted with ultrapure water into 25 mL volumetric flasks and then filtered into 50 mL conical flasks. Pb concentration was determined using an atomic absorption spectrometer (ZEEnit-700P, Analytikjena, Jena, Germany), which maintains the average measurement error within a relative error range of 0.1–5.0%, with a measurement range of ppm-ppb levels [30]. Quality control included blank samples and nationally certified reference materials of plant material (GBW10046a). The recovery of Pb in the standard plant samples was 103 ± 1.12% (85.0–115.0%), meeting the requirements of the national standard. All reagents used in the experiments were of ultra-pure grade, and the glassware and digestion tanks were soaked in an acid vat (concentrated nitric acid/water = 1:3) for 48 h. All samples were analyzed in triplicate.

#### 2.4.2. Subcellular Distribution of Pb (Differential Centrifugation Method)

A total of 0.50 g of wheat leaves was taken with 5 mL of pre-cooled extract (0.25 mol·L^−1^ sucrose, 50 mmol·L^−1^ Tris-HCl buffer, and 1 mmol·L^−1^ dithioerythritol) and ground in a mortar and pestle until it was homogenized. It was then transferred to a 50 mL centrifuge tube. The homogenate was first centrifuged at 3000 r·min^−1^ for 15 min to separate the precipitate (F1) from the supernatant, and then the supernatant was centrifuged at 15,000 r·min^−1^ for 30 min to separate the precipitate (F2) from the supernatant (F3). The F1 precipitate was mainly cell wall components, the F2 precipitate was mainly organelle components, and the F3 supernatant components mainly included cytoplasm, vesicles, and other cell sap components.

F1, F2, and F3 were added to 10 mL of HNO_3_-HClO_4_ solution, and the acidification reaction was carried out for 1 h. Then, they were transferred to 250 mL conical flasks and placed on an electric heating plate to start heating. Heating was carried out in a stepwise manner: firstly, the temperature was adjusted to 120 °C for 30 min, then increased to 180 °C for 30 min, and finally, the temperature was increased to 220 °C for digestion

#### 2.4.3. Extraction of Pb Chemical States

In order to determine the chemical state of Pb in wheat leaves, a stepwise extraction method can be used to separate different states of Pb using different extractants [31]. The specific steps were as follows: 2 g of fresh wheat sample was taken and cut. To it, 50 mL of extractant was added, and the sample was placed in a thermostat at 30 °C for 17–18 h. After recovering the extractant, the same volume of extractant was added again and placed in a thermostat for 2 h. The extraction was repeated four times within 24 h. The extract was then evaporated to near-dryness, digested, and analyzed for Pb concentration using an atomic absorption spectrometer (ZEEnit-700P, Analytikjena, Germany), and the analysis was repeated three times for all samples. The extractants used included (i) 80% ethanol, which mainly extracted Pb(NO_3_)_2_, PbCl_2_, and Pb in an amino acid state; (ii) deionized water, which mainly extracted water-soluble organic acid Pb; (iii) 1 mol·L^−1^ NaCl, which extracted pectin Pb and Pb in a protein-bound or adsorbed state, etc.; (iv) 2% ethylic acid, which mainly extracted Pb in a phosphate state, etc.; (v) 0.60 mol·L^−1^ hydrochloric acid, which mainly extracted oxalate Pb, etc. The residue fraction was mainly composed of silicate of Pb.

#### 2.4.4. SEM-EDS Observation

The elemental composition and stomata state of wheat leaves during the filling stage were observed using scanning electron microscopy (SEM) (JSM-7001F, Tokyo, Japan), with magnification ranging from 10 to 500,000 times, a secondary electron image resolution of 1.2 nm (at 30 kV), and a backscattered electron resolution of 3.0 nm (at 1 kV), and energy-dispersive spectroscopy (EDS) (NORANsystem7, Waltham, MA, USA), which has high-precision qualitative and quantitative analysis capabilities, with an elemental analysis range generally from boron (Be) to uranium (U)). After the wheat leaves were freeze-dried in a vacuum freeze-dryer (Beijing Boykang Experimental Instrument Co., Ltd., Beijing, China), they were cut into lengths of about 4 mm and placed on a copper base with conductive adhesive. The surfaces of the leaves were sprayed with gold for 180 s to improve their conductivity and secondary electron yield [32]. Finally, SEM was used to identify microscopic stomata and atmospheric particles in the images, and the surfaces of the leaves observed in the SEM images were analyzed using EDS to identify the weights and atomic percentages of the elements Pb, Cl, K, C, N, and O [32].

### 2.5. Data Analysis

#### 2.5.1. Calculation of Grain Filling Rate and Pb Accumulation Rate in Grains

The grain filling rate and Pb accumulation rate in grains can be calculated according to the following equations:

(1) $$G1=\frac{\Delta m}{\Delta t}$$

(2) $$G2=\frac{\Delta n}{\Delta t},n=m\times c$$

where *G*1 refers to the rate of grain filling (mg·1000 grains^−1^·d^−1^), *m* refers to the weight of wheat per thousand grains per period, ∆*m* refers to the increase in the quality of wheat per thousand grains per period (mg), and ∆*t* refers to the number of days between wheat sampling intervals (d). *G*2 is the rate of Pb accumulation in wheat grains (mg·1000 grains^−1^·d^−1^), and ∆*n* refers to the increase in Pb per thousand grains per period between wheat sampling intervals (mg); *c* refers to the mean Pb concentration of per thousand grains.

#### 2.5.2. Statistical Analysis

The significance of the differences in the data in terms of wheat yield and Pb concentration was evaluated by analysis of variance (ANOVA) and least significant difference (LSD) [33]. ANOVA can simultaneously compare the means of three groups and is suitable for research designs involving two treatment groups and a control group. Its assumptions include the normality of data distribution within each group and the homogeneity of variances between groups. After ANOVA shows overall significance, LSD can be used as a post hoc test to determine which groups have significant differences. The assumption of the ANOVA test is that there is no significant difference between the means of the groups, while LSD tests for significant differences between each pair of groups. The significance level for all analyses was set at 5% (*p* < 0.05). All data were analyzed in SPSS 20.0 (IBM Corp., Armonk, NY, USA) and expressed as mean ± standard deviation (S.D.) [34]. The insets were made using Origin 9.0 (OriginLab, Northampton, MA, USA) [35].

## 3. Results and Discussion

### 3.1. Effects of Pb Stress on Pb Concentration in Wheat Organs

**Figure 4:**
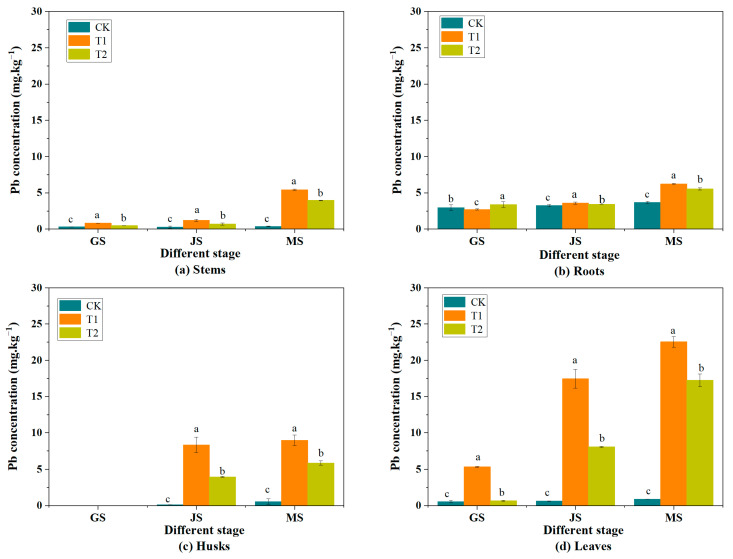
Pb concentration of different treatments in stems (**a**), roots (**b**), husks (**c**), and leaves (**d**) at different periods. (Note: Different letters in the figure (**a**–**d**) indicate that the differences are statistically significant (*p* < 0.05). Data are expressed as mean ± SD (n = 3). CK, T1, and T2 are control group, Pb(NO_3_)_2_ spray, and PbS spray, respectively. GS = greening stage (11 March); JS = jointing stage (29 April); MS = mature stage (1 June).

As the wheat grew, the Pb concentration in each organ of both treatment groups progressively increased with the duration of stress exposure. From GS to FS2, the Pb concentration in the roots, stems, and leaves of the T1 treatment group rose from 2.69 mg·kg^−1^, 0.80 mg·kg^−1^, and 5.32 mg·kg^−1^, respectively, to 6.21 mg·kg^−1^, 5.42 mg·kg^−1^, and 22.53 mg·kg^−1^. For the T2 treatment group, the initial Pb concentrations at GS were 3.37 mg·kg^−1^ in the roots, 0.45 mg·kg^−1^ in the stems, and 0.67 mg·kg^−1^ in the leaves, increasing to 5.53 mg·kg^−1^, 3.95 mg·kg^−1^, and 17.24 mg·kg^−1^ at FS2. Compared to GS, there was a significant increase in Pb concentration in the roots, stems, leaves, and grains of both treatment groups (*p* < 0.05). This is similar to the results obtained by Ma et al. [3], who conducted an experiment in areas with severe atmospheric Pb pollution and found that the Pb concentration in each organ of the wheat plant increased. However, the Pb concentration in each organ of the T2 group consistently remained lower than that of the T1 group. This may be attributed to the fact that the absorption of heavy metals by plants is contingent upon the metals’ state, environmental concentration, and solubility [36], and Pb(NO_3_)_2_ has a higher solubility and is more susceptible to translocation and bioavailable after penetrating the leaves than PbS. At FS2 and MS, the Pb concentrations in the roots, stems, leaves, husks, and grains of the T2 group were 89.1%, 72.8%, 76.5%, 65.4%, and 62.9% of those in the T1 group, respectively. In MS, the Pb concentration trend in the organs of the CK group was root > leaf > husk > stem > grain, whereas in the treatment group, it was leaf > husk > root > stem > grain (Figure 4). However, the results of Ma et al. [3] showed that the Pb concentration was highest in the roots, followed by the leaves, then the husks, and finally the grains. The reason for the discrepancy in results is that their experiments were conducted in areas with severe soil lead pollution. The cell walls of plant root cells are abundant in functional groups (-COOH, -SH, and -OH), which can react with Pb to form chelates and store them in the roots [29,37]. As a result, the migration of Pb to the aboveground environment is constrained, so most of the Pb absorbed by the roots from the soil is stored in the root system. In contrast, this experiment was conducted in an area with no Pb pollution, and only the leaves were subjected to Pb stress, which is why the Pb concentration in the leaves is higher than that in the roots. The Pb concentration in the leaves was significantly higher than that in other organs. The Pb concentration in the leaves of the T1 group was 4.1 times that in the stems and 3.6 times in roots, respectively, and in the T2 group, it was 4.3 times and 3.1 times that in the stems and roots, respectively. This disparity may reflect a plant defense mechanism aimed at minimizing the translocation of Pb from the leaves to other organs, thereby reducing the physiological damage caused by Pb to the wheat’s system. Similarly, the role of leaves as the main sink for Pb in plants is emphasized.

### 3.2. SEM-EDS Analysis

**Figure 5:**
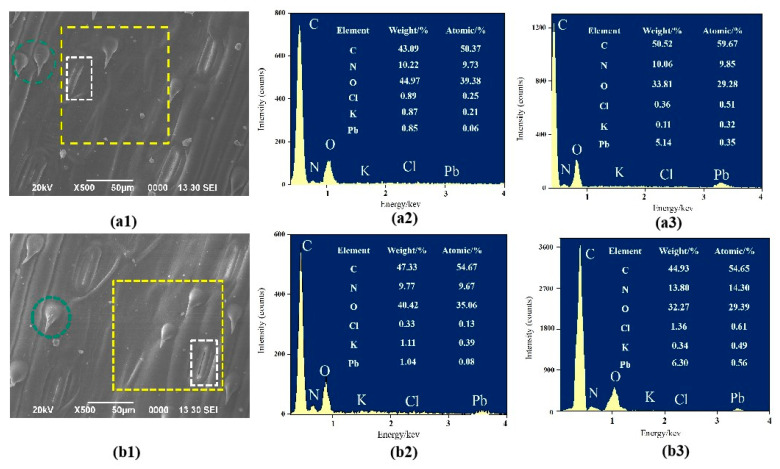
EDS analysis of stomata surface (T1, Pb(NO_3_)_2_ spray (**a1**–**a3**); T2, PbS spray (**b1**–**b3**)). The scanning area in (**a2**,**b2**) is shown by the yellow wire frame, and the scanning area in (**a3**,**b3**) is shown by the white wire frame; the magnification is 500×. The green areas show the trichomes.

To visually assess the interaction of wheat leaves with atmospheric particulates, we employed SEM-EDS to examine leaves from the T1 and T2 treatment groups during the filling period (Figure 5). The result indicated that the leaf surface is abundant in stomata, measuring approximately 25.25 to 28.53 μm in length and 5.76 to 7.52 μm in width, with the stomatal length notably exceeding the dimensions of PM_2.5_ and PM_10_ particles. It was evident that fine particles were adsorbed onto the leaf surface, with additional particles observed in the vicinity and even within the stomata (Figure 5(a1,b1)). EDS analysis confirmed the presence of Pb in these particles, with atomic percentages of 0.06 wt% and 0.08 wt% within the yellow wireframe areas (Figure 5(a2,b2)). Focusing the analysis on the white areas, the atomic percentage of Pb in the fine particles rose to 0.35 wt% and 0.56 wt% (Figure 5(a3,b3)), marking an increase of 0.29% and 0.48% compared to the yellow wireframe areas. These results suggest a higher concentration of Pb around the stomata and its accumulation within them, likely due to the ability of soluble Pb salts to penetrate the leaves through cuticular water pores and stomata, as well as the entry of water-insoluble Pb particles through stomata [38,39]. The green area indicates the trichomes, which, together with the cracks in the leaf surface cuticle, increase the roughness of the leaf surface, thereby promoting the accumulation of fine particles on the leaf surface [40]. This study discovered that as leaf stomata ingest fine particles, some become ensnared at the stomatal opening, impeding stomatal closure. Consequently, Pb-enriched fine particles can penetrate mesophyll cells through these gaping stomata in substantial quantities [15]. Stomata are an important pathway for leaves to absorb heavy metals and the only pathway for them to absorb a large number of solid particles with low solubility, and stomata openness is an important factor affecting the crop absorption of AMP-Pb [40]. The research of Gao et al. [32] suggests that Pb in PM_2.5_ can be translocated from APM to leaves via stomata, with large pore sizes accounting for elevated Pb accumulation in leaves. Moreover, Pb-laden particles can engage in plant metabolism and translocate to other organs via the phloem and xylem [5,41]. This aligns with the observation of Kumar et al. [41] that Pb distribution is evident in xylem, phloem, and mesophyll cells once Pb penetrates leaf tissue. Pb can also concentrate in phloem cells and translocate to the grain [42,43]. In conclusion, wheat leaves are capable of absorbing and translocating APM-Pb through stomata, leading to its accumulation in the grains.

### 3.3. Effects of Different Treatments on Subcellular Pb Distribution in Leaves

**Figure 6:**
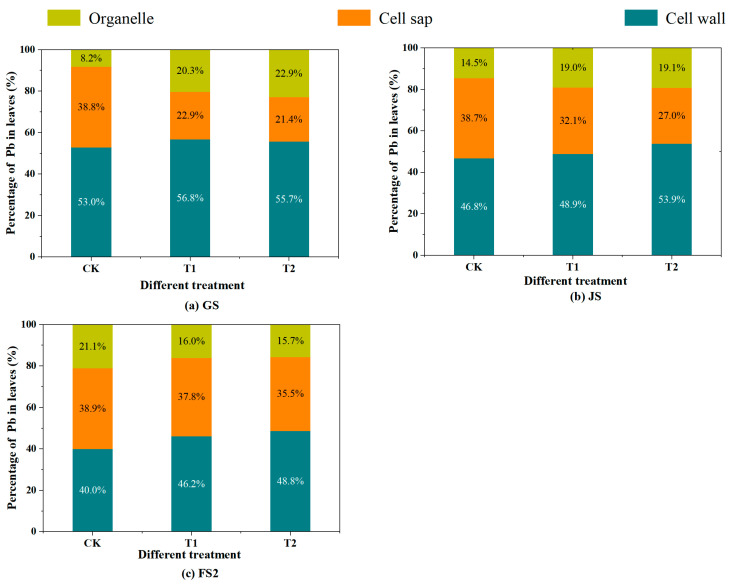
Subcellular distribution of Pb in wheat leaves at the greening stage (**a**), jointing stage (**b**), and late filling stage (**c**). (Note: CK, T1, and T2 are the control group, Pb(NO_3_)_2_ spray, and PbS spray, respectively. GS = greening stage (11 March); JS = jointing stage (29 April); FS2 = late filling stage (16 May)).

To investigate the subcellular distribution pattern of Pb(NO_3_)_2_ and PbS in leaf cells after penetrating the leaves, the leaves in the T1 and T2 treatment groups were centrifuged and digested. The results show that, in comparison with the GS, the Pb concentration in the cell wall, cell sap, and organelles of leaves at FS2 significantly increased following treatment with both Pb(NO_3_)_2_ and PbS (*p* < 0.05) (see Supplementary Materials for specific data). This phenomenon indicates that the accumulation of Pb in leaves is continuous with the progression of the growth stage, which is consistent with the research results of Ma et al. [3]. However, the concentration of the T2 treatment group is always lower than that of the T1 group, because the absorption of heavy metals by plants is known to be contingent upon the metals’ state, environmental concentration, and solubility [44]. Given that Pb(NO_3_)_2_ is considerably more soluble in water than PbS, it penetrates the leaf tissue in solution form through the cuticular water pores, cuticle cracks, and stomata. Conversely, PbS, being less soluble, primarily penetrates the leaf in a solid state via the stomata. Therefore, the Pb concentration of all organs in the T2 treatment was lower than that in T1. This differential uptake underscores the role of stomata in the internal translocation of atmospheric Pb. Across all growth stages, the subcellular distribution pattern of Pb in the leaves of both T1 and T2 treatments followed the order cell wall > cell sap > organelles (Figure 6), with Pb predominantly stored in the cell wall (46.2–56.9%), followed by the cell sap (21.4–37.8%). Notably, the proportion of Pb in the cell wall and organelles gradually decreased, while that in the cell sap increased, suggesting that the treatments diminished the cell wall’s retention capacity for Pb and enhanced the cell sap’s partitioning effect on Pb [45]. That is, with the intensification of Pb stress, Pb moves from the cell wall into the protoplast, transits to the cell sap, and accumulates in the vacuole, thereby safeguarding the normal physiological metabolism of other organelles [46,47,48]. This is because as the stress intensifies, the heavy metal binding sites on the cell wall tend to become saturated, forcing the Pb absorbed by the leaves to be transferred to other components. Additionally, vacuolar sequestration is another important detoxification mechanism in plants, which leads to an increase in the proportion of lead in the cell sap [49]. The specific cellular distribution of Pb can help alleviate the toxic effects of Pb on wheat plants. Figure 6 shows that the proportion of Pb in the cell wall for the T1 and T2 treatment groups decreased by 10.6% and 6.9%, respectively, from GS to FS2, while the proportion of Pb in the cell sap increased by 14.9% and 14.1%. The proportion of Pb in the cell sap fraction increases the most, indicating that the Pb entering the cell sap has the highest translocation capacity. Moreover, the Pb concentration in the grains shows an increasing trend, suggesting that Pb in the cell sap can be translocated to the grains through the phloem and xylem.

### 3.4. Chemical Extraction State of Pb in Wheat Leaves

**Figure 7:**
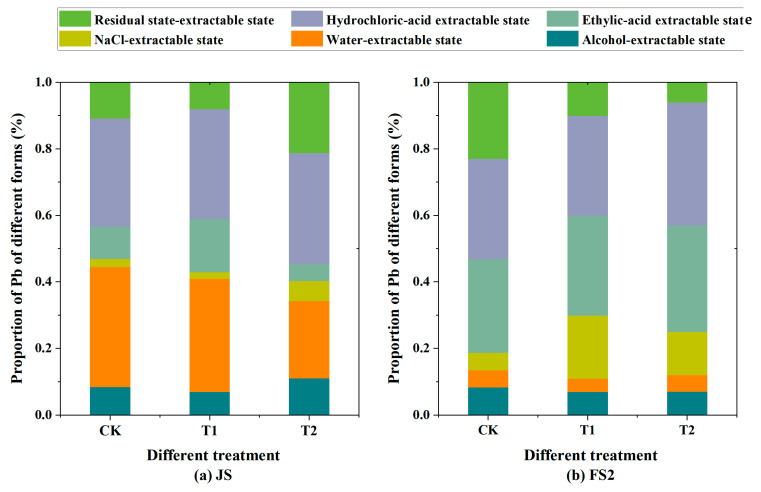
Distribution of chemical extraction states of Pb in wheat tissue at the jointing stage (**a**) and late filling stage (**b**). (Note: CK, T1, and T2 are control group, Pb(NO_3_)_2_ spray, and PbS spray, respectively).

The concentrations and relative proportions of various chemically extractable states of Pb in wheat leaf tissue are detailed in Table 1 and illustrated in Figure 7. At JS, for the T1 treatment group, the predominant state of Pb in leaf tissue was the highly active water-extractable fraction, which constituted 34.0% of the total. This was succeeded by the hydrochloric acid-extractable fraction, representing 33.0%, and the ethylic acid-extractable fraction, which accounted for 15.9%. By the full FS2, the proportions of the water-extractable and hydrochloric acid-extractable fractions of Pb had diminished to 4.0% and 30.0%, respectively, while the ethylic acid-extractable fraction had increased significantly, comprising 30.0% of the total Pb concentration. In the JS of the T2 treatment group, the hydrochloric acid-extractable state of Pb in leaf tissue was the most abundant, at 33.3%, followed by the water-extractable state at 23.2% and the residual state at 21.2%. The ethylic acid-extractable and NaCl-extractable states of Pb had nearly equivalent proportions. By FS2, the water-extractable state of Pb in the T2 group had decreased to 5.0%, the hydrochloric acid-extractable state had risen to 37.0%, and the ethylic acid-extractable state had increased markedly, accounting for 31.9% of the total Pb concentration.

The chemical form of Pb determines its bioavailability, toxicity, and potential for mobility. The T1 treatment group exhibited a higher proportion of both the less toxic and less mobile hydrochloric acid-extractable and ethylic acid-extractable states, as well as the more toxic and mobile ethanol-extractable and water-extractable states, compared to the T2 treatment group. This disparity may stem from the greater water solubility, mobility, and toxicity of Pb(NO_3_)_2_, facilitating more effective internal leaf penetration and a lower proportion of the residual state, whereas PbS behaves conversely. Thus, it is expected that the T1 treatment group would exhibit higher proportions of ethanol-extractable, water-extractable, and acid-extractable states than the T2 treatment group, which is supported by our results. Comparing FS2 with JS, both the water-extractable and ethanol-extractable fractions of Pb in the T1 and T2 groups were reduced by 30.0% and 22.3%, respectively. This suggests that highly active Pb species are likely translocated to other tissues and organs along with the filling material during the filling stage [38]. Despite differences in the proportions of components between the T1 and T2 treatment groups, the trend in the change in chemical extractable states was consistent between the two treatment groups, indicating that the chemical behavior of PbS and Pb(NO_3_)_2_ within the leaf tissue was analogous. Compared with JS, the concentration of water-extractable and ethanol-extractable fractions increased significantly, indicating that Pb in leaves at FS2 had more potential to translocate to the grain with the filling material.

### 3.5. Characteristics of Pb Accumulation in Wheat Grains at the Filling Stage

**Figure 8:**
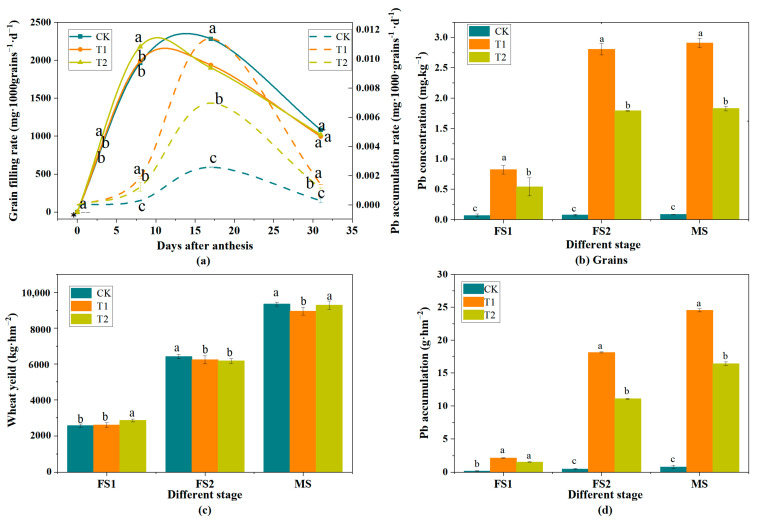
Grain filling rate and Pb accumulation rate (**a**), grain Pb concentration (**b**), wheat yield (**c**), and grain Pb accumulation (**d**) in wheat grains in different treatment groups at different filling stages. (Note: Different letters in the figure (**a**–**d**) indicate that the differences are statistically significant (*p* < 0.05). Data are expressed as mean ± SD (n = 3). CK, T1, and T2 are control group, Pb(NO_3_)_2_ spray, and PbS spray, respectively. FS1 = early filling stage (7 May); FS2 = late filling stage (16 May); MS = mature stage (1 June)).

As depicted in Figure 8, the grain filling rates for the T1, T2, and CK groups all exhibit a unimodal curve, characterized by an initial rise followed by a decline. The T1 and T2 treatment groups achieved their peak grain filling rates on the 7th day of the filling period, whereas the CK group reached its maximum at a later stage. After the CK group had reached its peak grain filling rate, the grain filling rates among the treatments followed the order CK > T1 > T2 (Figure 8a). This disparity can be attributed to the Pb spray treatment on wheat leaves, which diminishes leaf biomass, impairs chloroplast structure, and reduces chloroplast counts [50]. For instance, the research of Hou et al. [51] on *P. crinitum* indicated that chlorophyll a and total chlorophyll concentrations were significantly diminished in Pb-exposed plants compared to the control group. Despite the development of some resistance and chlorophyll degradation recovery in wheat as it matures, continuous Pb exposure amplifies stress, leading to a resurgence of Pb’s toxic effects on wheat. During the filling stage, this can adversely impact the filling rate. Consequently, the diminished grain filling rate is primarily due to the reduced photosynthetic capacity of the leaves, hindering the translocation of nutrients and Pb to the grains. This decline in photosynthetic capacity is attributed to both the toxic effects of Pb on leaves and the natural aging process of the leaves [44].

The accumulation rate of Pb in the grains mirrored this pattern, increasing initially and then decreasing, with the peak grain filling rate preceding the peak Pb accumulation rate. This suggests that Pb penetrates the grains with the filling material [44,52]. Throughout the filling period, the Pb accumulation rate in the grains followed the trend T1 > T2 > CK (Figure 8a). The accumulation rate of Pb in the grains tapered off from the middle of filling, a result of both the dilution of Pb concentration by photosynthate produced in FS1 leaves and the waning photosynthesis and filling rate in the later stages [21].

The concentration of Pb in wheat grains for both the treatment and control groups gradually increased with the progression of the filling process, consistently showing the trend T1 > T2 > CK. At FS1, the Pb concentrations in the grains for the CK, T1, and T2 treatments were 0.07 mg·kg^−1^, 0.82 mg·kg^−1^, and 0.53 mg·kg^−1^, respectively. At MS, the grain Pb concentrations were 0.08 mg·kg^−1^, 2.91 mg·kg^−1^, and 1.83 mg·kg^−1^, respectively. Compared to CK, the grain Pb concentration in the T1 and T2 treatments was significantly higher (*p* < 0.05) (Figure 8b). At MS, the Pb accumulation in the grains of T1 and T2 was significantly greater than that of CK (*p* < 0.05), with the T2 treatment’s grain Pb accumulation reaching 67% of the T1 treatment’s level.

As the filling process advanced, wheat yield increased, but the yields of both treatment groups were lower than that of CK, potentially due to the toxic effects of Pb on wheat. The extant literature has demonstrated that Pb-contaminated soil can lead to reduced rice yields [52]. In our study, while the Pb spray treatment did affect wheat yield, the impact was not markedly significant. The treatment could only moderately reduce wheat yield [53], a consequence of the withering and death of wheat leaves in later stages, rendering the wheat ears the primary photosynthetic organs. Concurrently, the influence of the two Pb treatments on Pb accumulation in wheat was considerably more pronounced than their effect on wheat yield. The Pb concentration and accumulation in wheat grains, which showed T1 > T2 > CK, indicate that both Pb(NO_3_)_2_ and PbS absorbed by the leaves can be translocated to the grain. The final yield, with CK > T2 > T1, suggests that Pb stress exerts a noticeable impact on wheat yield.

## 4. Conclusions

Pb with varying solubility penetrates into the leaves through different pathways, exhibits similar bioavailability and mobility, and can be translocated to the grains during the filling stage with filling materials, leading to Pb contamination in wheat grains. The amount of Pb absorbed by wheat leaves solely through the stomata accounts for 76.5% of the total absorbed through the cuticle and stomata, indicating that wheat leaves can directly absorb APM-Pb through the stomata and that the stomatal pathway is the primary pathway for leaf absorption of APM-Pb. The innovation of this study lies in using Pb with different solubilities to simulate the various pathways through which different components of atmospheric particulate matter-bound lead (APM-Pb) penetrate leaves. By making reasonable assumptions, this study conducts a detailed investigation into the role of the stomatal pathway in Pb absorption. The conclusions highlight the significance of the stomatal pathway in the absorption of APM-Pb by wheat leaves and reveal the impact of atmospheric Pb pollution on the quality of wheat grains, providing a theoretical basis for the control of Pb contamination in wheat grains at the leaf surface. This study not only enhances our understanding of the physiological processes of heavy metal absorption in crops but also has practical significance for agricultural practice. Based on the above conclusions, measures can be taken to control industrial emissions at the source, develop and plant wheat varieties with low stomata density and small stomata, and apply foliar barrier agents to reduce the risk of APM-Pb penetrating wheat leaves through stomata, thereby ensuring the quality of wheat grains. However, the results of this study may not be applicable to other crops, as genetic differences may affect stomatal structure and Pb absorption mechanisms. Future research can explore the genetic basis of wheat stomatal traits and targeted breeding programs.

## Figures and Tables

**Table 1:** Distribution of chemical extractable states of Pb in wheat at filling stage(mg·kg^−1^).

Stage	Treatment	Alcohol-Extractable State	Water-Extractable Sate	NaCl-Extractable State	Ethylic Acid-Extractable State	Hydrochloric Acid-Extractable State	Residual State-Extractable State	Total	Recovery Rate (%)
Jointing stage	CK	0.07 c	0.30 c	0.02 c	0.08 c	0.27 c	0.09 c	0.82 c	94.6
	T1	0.80 b	3.90 a	0.23 b	1.83 a	3.79 a	0.92 b	11.47 a	92.8
	T2	1.18 a	2.47 b	0.64 a	0.54 b	3.55 b	2.26 a	10.64 b	95.1
Late filling stage	CK	0.08 c	0.05 c	0.05 c	0.27 c	0.29 c	0.22 c	0.95 c	97.4
	T1	1.57 a	0.90 a	4.28 a	6.76 a	6.76 a	2.26 a	22.53 a	91.1
	T2	1.22 b	0.86 b	2.25 b	5.53 b	6.41 b	1.04 b	17.32 b	92.5

Note: Different letters in the table (a, b, c) indicate that the differences are statistically significant at different stages (*p* < 0.05). Data are expressed as mean ± SD (n = 3). CK, T1, and T2 are control group, Pb(NO_3_)_2_ spray, and PbS spray, respectively.

## Data Availability

The original contributions presented in this study are included in the article; further inquiries can be directed to the corresponding authors.

## Supplementary Materials

The following supporting information can be downloaded at: https://www.mdpi.com/article/10.3390/toxics13030185/s1, Table S1: Subcellular distribution of Pb in wheat leaves (mg·kg^−1^).
