# Supplementary material for: Stomata as the Main Pathway for the Penetration of Atmospheric Particulate Matter Pb into Wheat Leaves
Source: Toxics. 2025 Mar 1;13(3):185. doi: 10.3390/toxics13030185 (PMC11945984; doi:10.3390/toxics13030185)
Supplement: Supplementary file 1 [file toxics-13-00185-s001.zip › toxics-3395007-supplementary.pdf]

Supplementary Table S1. Subcellular distribution of Pb in wheat leaves ($mg \cdot kg^{-1}$)

Stage	Treatment	Cell wall	Cell sap	Organelle	Total	recovery (%)
Greening stage	CK	0.26c	0.19b	0.04c	0.49c	92.6
	T1	3.00a	1.21a	1.07a	5.28a	93.1
	T2	0.39b	0.15c	0.16b	0.71b	96.2
Jointing stage	CK	0.29c	0.24c	0.09c	0.62c	94.7
	T1	7.98a	5.24a	3.10a	16.32a	96.2
	T2	4.31b	2.16b	1.53b	8.00b	90.9
Late filling stage	CK	0.36c	0.35c	0.19c	0.90c	93.4
	T1	10.39a	8.50a	3.60a	22.49a	90.7
	T2	8.47b	6.16b	2.72b	17.35b	92.8

Note: Different letters in the table (a, b, c) indicate that the differences are statistically significant at different stage ($P < 0.05$). Data are expressed as mean ± SD (n=3). CK, T1 and T2 were control group, spray $Pb(NO_3)_2$ and spray PbS, respectively.
